# The nursing profession: a critical component of the growing need for a nuclear global health workforce

**DOI:** 10.1186/s13031-019-0197-x

**Published:** 2019-03-25

**Authors:** Tener Goodwin Veenema, Frederick M. Burkle, Cham E. Dallas

**Affiliations:** 1 National Academy of Medicine, Washington, DC USA; 2 Department of International Health, Nursing and Public Health, Johns Hopkins School of Nursing, Centre for Humanitarian Health, Johns Hopkins Bloomberg School of Public Health, Baltimore, Maryland USA; 3 Harvard Humanitarian Initiative, Harvard University & T.H. Chan School of Public Health, Cambridge, USA; 4 Woodrow Wilson International Center for Scholars, Washington, DC USA; 5 Department of Health Policy and Management, Institute for Disaster Management, University of Georgia, College of Public Health, Athens, USA; 6 Department of Emergency Medicine, Clinical Professor of Emergency Medicine, Medical College of Georgia, Augusta University, Augusta, USA

**Keywords:** Nuclear weapons, Nuclear war, Nurses, Global health workforce

## Abstract

**Background:**

Instability in the global geopolitical climate and the continuing spread of nuclear weapons and increase in their lethality has made the exchange of nuclear weapons or a terrorist attack upon a nuclear power plant a serious issue that demands appropriate planning for response. In response to this threat, the development of a nuclear global health workforce under the technical expertise of the International Atomic Energy Agency and the World Health Organization Radiation Emergency Medical Preparedness and Assistance Network has been proposed.

**Main body of the abstract:**

As the largest component of the global healthcare workforce, nurses will play a critical role in both the leadership and health care effectiveness of a response to any public health emergency of international concern (PHEIC) resulting from the unprecedented numbers of trauma, thermal burn, and radiation affected patients that will require extensive involvement of the nursing professional community.

**Short conclusion:**

Lives can and will be saved if nurses are present. The clinical care of radiation contaminated patients (e.g. radiation burns, fluid management, infection control), thermal burn patients, and other health system response activities such as community screening for radiation exposure, triage, decontamination, administration of medical countermeasures and the provision of supportive emotional and mental health care will be overwhelmingly nurse intensive.

## Background

Despite low level awareness on the part of the public, concerns for the use of nuclear warfare against the United States dating back to the Cold War are now steadily increasing [1]. The National Security Strategy states that the American people face no greater or more urgent danger than a terrorist attack using a nuclear weapon [2] and in 2017 the Science and Security Board warned: “World leaders are failing to act with the speed and on the scale required to protect citizens from the extreme danger posed by climate change and nuclear war. The probability of global catastrophe is very high, and the actions needed to reduce the risks of disaster must be taken very soon” [3]. Given the heightened geopolitical tensions between countries in possession of nuclear weapons, the need for a health care workforce with the specific knowledge, skills and abilities to respond to a nuclear PHEIC is of critical importance. The intentional release of radiation will unquestionably create a substantial and potentially devastating burden upon a region’s health care system, and as such, on a region’s healthcare workforce. Plans for U.S. medical response have been described previously [4–6], and recognizing that “a nuclear event anywhere is a nuclear event everywhere”, in 2015, Burkle and Dallas proposed a framework for developing a nuclear global health workforce [7]. Nurses constitute the largest sector of the global healthcare workforce (2,955,200 active registered nurses in the U.S. healthcare workforce alone [8], and their capacity and willingness to respond to a radiation/nuclear event will be critical to the success of the health response [9]. The clinical care of radiation contaminated patients (e.g. fluid management, infection control), and other health system response activities such as community screening for radiation exposure, triage, decontamination, administration of medical countermeasures and the provision of supportive emotional and mental health care will be overwhelmingly nurse intensive [10, 11]. In addition, the mass casualty care required for the extensive thermal burn patients anticipated from any nuclear weapons event will also be very nurse response intensive, and especially so for the strikingly large number that would result from the recent rapid increase in the threat of thermonuclear war. The ubiquitous threat of a nuclear attack is real and the participation of a radiation competent nursing workforce will be imperative for an effective response. This paper presents the potential impact of a nuclear event on individual and population health, and positions nurses will serve as the foundation for a nuclear global workforce.

## Main text

Detonation of a nuclear device, especially in crowded urban areas as currently anticipated, will produce unprecedented numbers and kinds of injuries requiring a healthcare response not currently available anywhere in the world. The only experience with urban nuclear detonations, fortunately, has been with the bombs dropped on Hiroshima and Nagasaki, Japan at the end of World War II., While these weapons (10-15kT) were far greater in destruction and healthcare impact than all conventional weapons previously, in comparison to thermonuclear weapons now proliferating globally these were relatively small. Indeed, after two decades of preparing for a 10kT weapon detonation by DHS, FEMA has announced and presented at a recent meeting of the National Academy of Sciences that they are now focusing on 10-1000kT nuclear detonation response [12, 13].

### Healthcare response needs due to nuclear detonation

When a nuclear weapon explodes, it generates a blast wave, intense light and heat, radiation, and a large fireball is created (creating the characteristic mushroom-shaped cloud). Fallout is composed of fission-created radioactive elements which attach to vaporized debris particles from the explosion and then are carried by wind up to many miles from the site of the explosion. Detonation of a nuclear weapon would cause great destruction, death, and injury and have a wide area of impact. Individuals close to the blast site could experience injury or death from the blast wave, moderate to severe thermal burns from heat and mass fires, full or partial blindness from the intense light, and acute radiation syndrome or ARS (caused by the radiation released at the time of detonation). Individuals farther away from the blast, but in the path of fallout, may experience health effects from fallout on the outside of the body or clothes (external contamination) or on the inside of the body (internal contamination), from contaminated food, air, water sources, or contact with contaminated surfaces. Extensive modeling has been done of a potential detonation of a nuclear weapon in various locations [14, 15] with all studies indicating the daunting task for medical response.

### Failure to plan for nuclear events

A nuclear detonation anywhere in the world would have devastating results resulting in a PHEIC and there would be limited time to take critical protection steps. Social disruption, chaos and panic will ensue. In the immediate aftermath of a nuclear event many people will die, however the possibility exists that the great majority of people in most large cities experiencing a nuclear detonation would survive. Despite the fear surrounding such an event, emergency planning and preparation can lessen deaths and illness and research supports that lives can be saved if a rapidly deployed and robust multidisciplinary response component exists [1]. Public health and the acute health care system will play a key role in responding to the affected population. Yet an overwhelming sense of fatality and doom balanced with societies’ collective denial regarding the potential for use of nuclear weapons limits health care systems planning for a nuclear attack. A WHO report published in 1984 stated that the immediate and delayed loss of human and animal life would be enormous and “the plight of survivors would be physically and psychologically appalling.” This negative outcome is particularly applicable to the use of thermonuclear weapons (>50kT) in urban areas, though the outcomes for the smaller, Hiroshima-sized weapons is much less in magnitude, especially in the thermal burn casualty category, often described as the “Achilles heel” of nuclear war medical response. With the increasing concern tied to the recent spread of thermonuclear weapons to potentially aggressive nations (such as North Korea), this sense of fatality and doom which has helped preclude nuclear war medical response in the past is only likely to be even more of an issue in hampering rationale preparation that is indeed feasible.

### Nursing’s role in a nuclear response

A nuclear event will result in an unprecedented mass casualty incident with large scale morbidity and mortality, requiring a massive medical response [16], allocation of scarce resources and the rapid deployment of mobile, self-contained, self-sufficient health care facilities [15]. According to the nuclear workforce framework put forth by Burkle and Dallas, medical support to triage, provision of care to those with the opportunity to survive, palliative care for those who will die, and care for those individuals less affected or who have evacuated will be needed [1]. In recognition of the potentially thousands of people exposed, health care facilities (both mobile and fixed) will need to be rapidly established beyond existing emergency departments to meet the massive surge in demand for care. These nuclear health care settings will facilitate initial triage and dose-monitoring, assessment, decontamination, patient transfer, and provide access to definitive care. In order to deliver these services the framework proposes the establishment of 1) nuclear triage centers, 2) nuclear survival centers, 3) nuclear palliative care centers, and 4) health system support centers. Additionally, time-constrained radiation medical countermeasures will need to be rapidly deployed and administered to appropriate populations and mass sheltering may be needed for large numbers of evacuees. In each of these endeavors and across all of these settings, nurses will be needed to establish, sustain operations and provide initial and ongoing patient care (Table 1).

**Table 1:** Nursing’s Role in A Nuclear Response

Field-Based Centers Under the Nuclear Global Health Workforce^a^ and U.S. Public Health Response	Nurse Roles & Responsibilities	Nurse Professionals
Nuclear Triage Centers/Community Reception Centers	Medical triage using “fast biological dosimetry” Initial medical stabilization Exposure vs Contamination-Decontamination Thermal vs Radiation Burn Assessment Peer education and radiation exposure mitigation (principles of working with radioactivity, appropriate use of PPE, etc.) Surveillance and data collection Psychosocial support Health education regarding self-decontamination Coordination of patient transfers Interdisciplinary collaborative practice with Radiation safety officers, physicians, EMS and emergency managers	RNs Occupational health nurses Nurse Practitioners (psychiatric/mental health NPs, acute care/trauma NPs, primary care NPs would all have different, but valuable roles to fill in initial triage)
Point-of-Distribution Clinics (PODs) for Rapid Radiation Medical Countermeasures Deployment	Establish and staff PODs Screening and assessment Radiation (protective) medical countermeasure administration Patient monitoring Interdisciplinary collaborative practice with State Strategic National Stockpile (SNS) Coordinators, Pharmacists, EMS and emergency managers	RNs School nurses Public Health nurses Occupational health nurses LPNs/LVNs
Nuclear Survival Centers	Secondary triage (biodosimetry/bioassay) Hospital-level unit staffing Isolation staffing Pain and symptom management Burn care- Initial assessment and stabilization, fluid/electrolyte management, infection control, debridement, nutrition support Psychosocial support Spiritual and culturally sensitive care of patients and their families Family Reunification	Acute and chronic care nurses and Nurse Practitioners (surgical nurses, burn nurses, oncology nurse, emergency and critical care nurses) Nurse Anesthetists Psych/Mental Health Nurses and Nurse Practitioners Infection Control nurses Occupational health nurses
Nuclear Palliative Care Centers	Pain and symptom management End-stage burn/acute radiation syndrome care Psychosocial support Spiritual and culturally sensitive care of patients and their families Patient education and advocacy Ethical and legal considerations Interdisciplinary collaborative practice with physicians, pharmacists, family counselors and social workers, clergy Loss and grief, bereavement care Engagement of community resources for family support post death	Hospice and palliative care nurses and nurse practitioners Primary care nurses and nurse practitioners Nurse anesthetists Psych/mental health nurses and nurse practitioners Parish nurses LPNs/LVNs
Health System Support Centers	Hospital/clinic/mobile facility staffing Rehabilitation Care of displaced, evacuated patients and families Patient education and advocacy Ethical and legal considerations Family Reunification Psychosocial support Spiritual and culturally sensitive care of patients and their families	Nurse Administrators Hospital and ambulatory clinic nurses Surgical nurses, burn nurses, oncology nurses Primary care nurses and nurse practitioners Rehabilitation Nurses Public Health nurses Psych/mental health nurses Occupational health nurses
Public Shelters	Temporary housing Feeding/Nutrition Safety/security Provision of essential supplies Child/infant care Infection control Population surveillance monitoring Psychosocial support Family reunification Manage volunteers Collaboration with non-governmental organizations (NGOs)	RNs Public Health Nurses LPNs/LVNs

^a^Burkle, F. M., & Dallas, C. E. (2016). Developing a nuclear global health workforce amid the increasing threat of a nuclear crisis. *Disaster medicine and public health preparedness*, *10* [1], 129–144

### Nuclear triage centers/community reception centers

Screening programs will need to be implemented as an immediate primary public health response given the large numbers of people potentially exposed to radioactive fallout, a significant number of whom will have radioactive particles on their clothes and on their person. In addition to providing the much needed screening, this will also divert large numbers of uninjured people away from the primary health delivery facilities, which would otherwise be inundated with these people who are likely to be highly motivated by fear to seek assistance. Crowding with seriously injured patients will already be bad enough, and taking the pressure off these critical facilities is highly important. Nurses will be needed to staff these nuclear triage centers as members of the radiation exposure screening and population monitoring team in conjunction with radiation safety experts and other health care providers. In the United States, the Centers for Disease Control and Prevention (CDC) advocates establishing Community Reception Centers (CRCs) in the aftermath of a sufficiently large radiologic event- a similar concept to the nuclear triage centers (NTCs). The purpose of these NTCs/CRCs will be to provide radiologic screening for uninjured or lightly injured people, to provide decontamination when necessary, and to refer those with likely internal contamination on for radiologic assessment and immediate medical countermeasure administration. This referral of verified internally contaminated patients, as well as patients with high estimates of external radiation exposure, will also be an essential function for decision-making of the use of radiation related pharmaceuticals from the Strategic National Stockpile (SNS). Patients (potentially thousands) will present with varying degrees of radiation exposure including acute radiation syndrome (ARS), local radiation injuries, and radiation combined, burn and blast (ocular, ear and lung) injuries. Nurses will assist with ‘fast biological dosimetry’ initial triage to determine “probability of fatality” [17] or implement the Exposure and Symptom Triage Tool (EAST) for rapid assessment of radiation exposure [18]. Nurses will conduct secondary and serial triage to provide ongoing assessment of severity of injury and other clinical issues. Nurses will interpret triage assessments, implement clinical guidelines [19] and coordinate patient transfers and care as scarce resources (such as the SNS) become available [20].

### Point-of-distribution clinics for rapid medical countermeasures deployment

Medical countermeasures, or MCMs, are FDA-regulated products (biologics, drugs, devices) that may be used in the event of a potential public health emergency stemming from a terrorist attack with a biological, chemical, or radiological/nuclear material, or a naturally occurring emerging disease. Recently, the number of MCMs for use following nuclear events has been significantly expanded, due to the increasing threat. Radiation mitigators are drugs administered shortly after radiation exposure that accelerate recovery or repair of radiation injury. Radionuclide eliminators are drugs that decorporate or block absorption of internalized radionuclides and include potassium iodide (KI), Prussian blue (PB), and zinc/calcium diethylenetriamine pentaacetate (Ca- and Zn-DTPA). In the immediate aftermath of a nuclear event specific populations may benefit from rapid access to radiation MCMs in the NTCs/CRCs. Some high dose internally contaminated patients would be selected to receive the radionuclide eliminators that flush the radionuclides out of the body or block absorption. Other patients with high doses received from internal and/or external radiation might be selected to receive the radiation mitigators, especially those determined to need amelioration of the effects of radiation-induced bone marrow depression (i.e. low white blood cell counts). Nurses would be essential in each stage of this process of patient evaluation and decisions on the distribution of limited availability of these highly specialized pharmaceuticals. In order to mobilize and distribute these MCMs, nurses will be needed to establish and sustain point-of-distribution clinics (PODs), screen patients and determine eligibility, council patients regarding potential side effects, and to administer the MCMs. Nurses will be needed to care for patients who have adverse medication events and to facilitate medical follow-up.

### Nuclear survival centers

Nuclear survival centers will be needed to accommodate the surge of patients requiring higher level clinical care and to help ‘decompress’ the overwhelming burden placed upon existing hospitals and emergency departments. The design may include both fixed and mobile hospital-based facilities to optimize survival opportunities for victims and to mitigate secondary indirect morbidity and mortality [1]. Nurses will be a major personnel necessity in nuclear survival centers to continue the triage process, conducting secondary triage (biodosimetry/bioassay) and to perform serial patient assessments. Nurses will provide hospital-level unit staffing and staffing of isolation rooms. Acute and chronic care nurses and nurse practitioners, particularly emergency, surgery, burn and critical care nurses will be needed for immediate stabilization of patients, render definitive care and to facilitate patient movement through the continuum of care. There is already considerable concern over the steady decrease in training for burn treatment nurses in “normal” times. The dramatic surge in thermal burn cases expected with any nuclear weapon use, and the geometrically larger number of thermal burn cases with thermonuclear weapons is likely to be translated to a severe staffing shortage in qualified burn nurses that must be addressed. These nurses would provide thermal burn management including initial assessment and hemodynamic stabilization, fluid/electrolyte rescue and management, infection control, debridement, nutrition support and emotional support. Nurse anesthetists, psych/mental health nurses and nurse practitioners, oncology nurses, infection control nurses and occupational health nurses will provide pain control and symptom management, psychosocial support, and spiritual and culturally sensitive care of patients and their families.

### Nuclear palliative care centers

Responses to disasters and large scale humanitarian emergencies rarely include palliative care, the discipline devoted to preventing and relieving suffering rather than to specific diseases, organs or technical skills. In fact, a stark and somewhat false dichotomy exists between saving lives and relieving suffering. In the case of a nuclear event, the provision of palliative care will be extremely relevant to health care systems. This will be true for not only the immediate triage category, but also with the expectant group (alive during the healthcare crisis but not expected to survive). Triage decision making with the likelihood that resources such as pharmaceuticals will be far less than the patient population urgently needing them will require distinctive training on the part of the nurses facing these impending nuclear crises. Hospice and palliative nurses focus exclusively on end-of-life care and “help patients achieve the best possible quality of life through relief of suffering, control of symptoms, and restoration of functional capacity, while remaining sensitive to personal, cultural and religious values, believes and practices”.^1^ Palliative nursing care is the “comprehensive management of the physical, psychological, social, spiritual, and existential needs of patients, particularly those with incurable, progressive illness and has an important role in humanitarian crises [21]. Provision of clinical care in nuclear palliative care centers will require 24-h nursing availability, possibly for months, to anticipate and meet the needs of radiation affected patients and families facing terminal illness and bereavement. Pain and symptom management, end-stage burn and acute radiation syndrome care, psychosocial support and patient education and advocacy are just a few of the many nursing roles and responsibilities that will be needed in this setting. Primary care nurses and nurse practitioners, Nurse Anesthetists, Psych/mental health nurses, Parish nurses and LPNs/LVNs will be needed to collaborate with physicians, social workers, or chaplains within the context of an interdisciplinary team. Nurses will be needed who understand how to engage community resources for family support and burial services post death, and to work with clergy to help families address loss and grief and bereavement care.

### Health system support centers

Health system support centers are settings were populations in unaffected locations and evacuees can seek supplemental medical care and screening for noncommunicable diseases and other health issues. These settings support or restore public health and health care systems and may provide additional bed capacity and additional sources of care. Fixed or mobile facilities will need nurse staffing to provide care and rehabilitation for displaced, evacuated patients and their families. Nurse administrators, hospital and ambulatory clinic nurses, surgical nurses, burn nurses, oncology nurses, rehabilitation nurses and primary care nurses and nurse practitioners will be needed. Public health nurses, psych/mental health nurses and occupational health nurses will conduct population surveillance and monitoring, patient education and advocacy, address ethical and legal considerations in the provision of clinical care, and provide assistance with family reunification and psychosocial support.

### Public shelters

Public shelters differ from the clinical settings listed above and will have distinct and complementary operations focused primarily upon non-affected evacuees. Shelters will provide temporary housing, security, food service, and ongoing health surveillance to displaced populations [22]. They are not designed for the provision of medical and nursing care beyond first aid and care of minor illness. Ideally, evacuees will have processed through a NTC/CRC prior to arriving at a shelter, however with the panic likely to occur with nuclear weapons detonations of any size this may not be the case and special considerations will need to be in place to ensure the health and safety of health care providers and shelter residents. Nurses will be needed to establish and sustain shelter operations including the establishment of a shelter floor plan with detailed procedures for managing potentially contaminated people, securing contamination control zones and decontamination facilities. Baseline radiation levels will need to be ascertained and monitored and decisions made on the degree of decontamination necessary. As most radiation decontamination is likely to be highly effective with simply disrobing and safe disposal of contaminated clothing (as opposed to decontamination requiring unnecessary labor and other resources in an already taxing environment), decision making by properly trained nurses will be essential. Nurses will work with shelter radiation safety officers, local emergency managers and volunteers to accommodate the needs of the residents and to monitor the living, eating and sanitary spaces in the shelter. Shelters have food preparation and food service areas, restrooms, showers and infant care areas. Public health follow-up and monitoring of shelter residents, implementation of infection control measures, care and monitoring of pregnant women exposed to radiation will be done by nurses. Nurses trained in psychological first aid can assist evacuees with the psychological effects of surviving a nuclear event, educate evacuees about radiation risk and help with the transition back to home.

### Management of Psychosocial Crisis

The psychological, emotional and behavioral consequences of any nuclear weapon event are certain to be of staggering proportions, rippling through communities both near and far. Depression, anxiety, acute and post-traumatic stress disorder, poor self-reported health status and medically unexplained somatic symptoms characterize the psychological impact of large-scale radiation events [23]. The lifetime prevalence of depression in women 11 years after Chernobyl was double the lifetime prevalence in women in the Ukraine [24]. Fear of developing cancer may be long-lasting and perpetuate negative mental health impacts, leading to self-medication through the increased use of alcohol and pharmaceuticals, and an increase in family disintegration and violent/antisocial behavior. Social decay and civil unrest may occur. Nurses will be needed across diverse clinical settings and all sectors of society to assist individuals, families and communities to heal and move towards restoration of daily life in a ‘new normal’ post nuclear event.

### Challenges for workforce development

The detonation of a nuclear device whether in a U.S. city or anywhere in the world will create a PHEIC and a global need for nurses and other health care providers to manage casualties exposed to radiation, sustain the health care systems needed for response, and provide targeted clinical care. However, few nurses have either training or experience in the field of radiation injury [25]. Multiple factors will influence the capacity, capabilities and willingness of nurses to participate in a national or global response to a nuclear event. Nurses’ perception of their personal risk related to radiation exposure, their knowledge, abilities and skills, and their sense of clinical competence may impact the speed and integrity of the response [26]. A lack of clarity regarding nurses’ specific roles and responsibilities in the aftermath of a nuclear event and a lack of awareness or knowledge of clinical guidelines adds complexity to preparedness. Currently, 75 % of U. S Schools of Nursing do not include radiation/nuclear content in their programs of study basically ensuring that the next generation of nurses will be inadequately prepared [26, 27].

Using the proposed framework for a global nuclear workforce a tiered model of professional nursing practice is proposed to directly align to the anticipated roles and responsibilities for nurses in the event of a nuclear event. The Model of nursing practice for nuclear response addresses both clinical and health systems management (administrative) practice, from which programs of education and opportunities for training can be targeted to those sectors of the profession who would most benefit.

### Model of nursing practice for nuclear event response

- Tier 1: Any staff/clinic or public health nurse or advanced practice nurse who has completed a program of basic, generalized nursing education and is authorized to practice by the regulatory agency of his/her country. These nurses will need a baseline understanding of radiation concepts, population health effects, basic decontamination and the appropriate use of PPE.
- Tier 2: Any practicing nurse or advanced practice nurse who has achieved the Tier 1 radiation/nuclear competencies (especially thermal burn treatment) and is designated a disaster responder within an institution, organization or system, or deployed to a satellite clinical setting. These nurses need an advanced knowledge base including clinical care of acute radiation syndrome, management of radiation and thermal burns, radiation triage, and health systems management skills to establish and sustain the fixed or mobile satellite clinical care settings described above.
- Tier 3: Any nurse or advanced practice nurse who has achieved Level 1 and 2 radiation/nuclear competencies and is prepared to respond as a member of a deployable radiation rapid response team, or to serve as a fixed or mobile ‘base camp’ nuclear subject matter expert nurse advisor. These nurses need advanced knowledge of national/international nuclear response plans, crisis leadership skills and abilities and knowledge of health systems optimization strategies. These nurses will most likely assume clinical supervisory and systems leadership positions.

## Conclusions

The participation of a radiation competent nursing workforce will be imperative for an effective response to a PHEIC resulting from a nuclear event. Lives can and will be saved if nurses are present. The clinical care of radiation contaminated patients (e.g. radiation burns, fluid management, infection control), thermal burn patients (which are difficult even with small numbers), and other health system response activities such as community screening for radiation exposure, triage, decontamination, administration of medical countermeasures and the provision of supportive emotional and mental health care will be overwhelmingly nurse intensive. Policymakers, nursing educators, public health and health care systems administrators who include the profession of nursing as the foundational element in nuclear response plans will increase the probabilities of survival following these devastating events.

## Footnotes

1 Journal of Hospice and Palliative Care. Retrieved from https://www.nursesource.org/hospice.html.
